# Mu-opioid receptor-expressing neurons in the paraventricular thalamus modulate chronic morphine-induced wake alterations

**DOI:** 10.1038/s41398-023-02382-w

**Published:** 2023-03-03

**Authors:** Darrell Eacret, Elisabetta Manduchi, Julia Noreck, Emma Tyner, Polina Fenik, Amelia D. Dunn, Jonathan Schug, Sigrid C. Veasey, Julie A. Blendy

**Affiliations:** 1 Department of Systems Pharmacology and Translational Therapeutics, Perelman School of Medicine, University of Pennsylvania, Philadelphia, PA USA; 2 Department of Genetics and Institute for Diabetes, Obesity, and Metabolism, Perelman School of Medicine, University of Pennsylvania, Philadelphia, PA 19104 USA; 3 Center for Sleep and Circadian Neurobiology and Department of Medicine, Perelman School of Medicine, University of Pennsylvania, Philadelphia, PA 19104 USA

**Keywords:** Molecular neuroscience, Psychiatric disorders

## Abstract

Disrupted sleep is a symptom of many psychiatric disorders, including substance use disorders. Most drugs of abuse, including opioids, disrupt sleep. However, the extent and consequence of opioid-induced sleep disturbance, especially during chronic drug exposure, is understudied. We have previously shown that sleep disturbance alters voluntary morphine intake. Here, we examine the effects of acute and chronic morphine exposure on sleep. Using an oral self-administration paradigm, we show that morphine disrupts sleep, most significantly during the dark cycle in chronic morphine, with a concomitant sustained increase in neural activity in the Paraventricular Nucleus of the Thalamus (PVT). Morphine binds primarily to Mu Opioid Receptors (MORs), which are highly expressed in the PVT. Translating Ribosome Affinity Purification (TRAP)-Sequencing of PVT neurons that express MORs showed significant enrichment of the circadian entrainment pathway. To determine whether MOR + cells in the PVT mediate morphine-induced sleep/wake properties, we inhibited these neurons during the dark cycle while mice were self-administering morphine. This inhibition decreased morphine-induced wakefulness but not general wakefulness, indicating that MORs in the PVT contribute to opioid-specific wake alterations. Overall, our results suggest an important role for PVT neurons that express MORs in mediating morphine-induced sleep disturbance.

## Introduction

Opioids contribute to dysregulated sleep, and the majority of people with Opioid Use Disorder (OUD) report sleep problems [1–3]. OUD patients that have sleep problems show increased craving and increased risk for relapse [4, 5]. In humans, morphine and methadone reduce slow wave sleep (stage 3–4 NREM), increase stage 2 sleep, and decrease percent time in REM sleep compared with baseline and placebo controls [6, 7], shifting the sleep architecture towards lighter stages of sleep. These types of objective studies in healthy subjects do not exist for patients on chronic opioids or with OUD, but correlational studies support the concept that individuals maintained on opioids have sleep disruption. Based on the Pittsburgh Sleep Quality Index (PSQI), 80% of individuals dependent on prescription opioids had poor sleep quality compared to 8% of the control group, and 41% of individuals in the prescription opioid group reported sub-threshold, moderate, or severe insomnia compared to 12% of the control group [8]. The prescription opioid group also had significantly increased sleep latencies, decreased sleep efficiency, decreased total sleep time, and increased total time awake based on an actigraphy device [8]. The majority of subjects taking over 100 mg methadone reported sleep problems based on the PSQI [9]. In patients prescribed opioids compared to a control group, 93% of the participants with a PSQI over 5 (poor sleep) were from the opioid group, and 83% of the patients that marked “excessively sleepy during the day” were from the opioid group [10]. The vast majority of patients that had 180 mg of morphine per day have either obstructive sleep apnea, central sleep apnea, or both [11]. Opioid-abstinent patients show increased craving and risk for relapse, and a subset of patients even cite sleep problems specifically as a reason for relapse back to opioids [5, 12]. Sleep and opioids appear to have a fundamental neurobiological association, resulting in a detrimental feedforward cycle. Understanding and targeting sleep symptoms in the context of opioid use could contribute to more effective abstinence.

Morphine binds potently to the mu-opioid receptor, and variances in the gene encoding the mu-opioid receptor (*OPRM1*) can affect sleep during opioid use. In a study of individuals prescribed 3 months of opioids for chronic noncancer pain, patients that had the single nucleotide polymorphism (SNP; rs1799971- A118G) for the minor allele 118-GG had decreased sleep adequacy scores and increased self-reported sleep problems in the SLP-9 index compared to patients with the 118AA or 118 AG genotypes [13]. In a separate study, 18% of patients on methadone maintenance therapy (MMT) were categorized as having insomnia (those that had higher doses of methadone had increased severity of insomnia), and 12 OPRM1 SNPs were significantly associated with increased insomnia scores [14]. This finding of ORPM1 SNPs decreasing sleep quality for patients on MMT was supported by another study which found that opioid-dependent MMT patients with the *OPRM1* AC/AG diplotype (A at 118 and C or G at 691) had lower PSQI scores, indicating better sleep, showing additional polymorphisms in the *OPRM1* gene contribute to sleep quality [15]. Taken together, these studies are consistent with the idea that the mu-opioid receptor can regulate opioid-related sleep and that variation in the OPRM1 gene is associated with worse quality of sleep, particularly when individuals are taking opioids.

In mice, sleep or sleep-regulating neurons provide a link between reward, drugs of abuse, and sleep. Specifically, cocaine craving [16] withdrawal [17], and sucrose but not food seeking [18] all engage sleep-regulating neurons. Morphine increases wakefulness in a dose-dependent manner while decreasing NREM and REM sleep, and higher doses of morphine eliminated REM sleep in a 4-h period [19]. Subcutaneous injections of morphine increase wake and decrease NREM and REM in a 3-h period in rats [20, 21]. Both acute and chronic fentanyl increase wakefulness and decrease NREM time in mice, and the same effect was exacerbated in mice lacking the circadian transcription factor NPAS2 [22]. Morphine administration during the light cycle but not the dark cycle increases the firing frequency of PVT neurons, indicating a circadian-dependent response to morphine in the PVT [23]. PVT neurons themselves bidirectionally control sleep/wake behaviors [24]. Neurons in the PVT project to the NAc in a pathway that is necessary and sufficient to control the expression of somatic signs of opiate withdrawal in mice [25]. The PVT controls salience and associative learning for both positive and negatively valenced stimuli, including reward [26]. The PVT is also involved in mediating opiate-assisted memories, as PVT projections to the central amygdala control morphine-related memory formation, while PVT projections to the NAc are important for morphine-associated memory retrieval and relapse [27]. Given the PVT’s role in sleep, circadian rhythms, and response to opioids, we sought to test the specific function of PVT MOR- expressing neurons in morphine-induced sleep disruption.

## Methods

### Mice

All studies were performed at the University of Pennsylvania and were in accordance with the Institutional Animal Care and Use Committee policy. Male and female mice on a C57Bl/6J background were used in all studies. Mice were given ad libitum food and were maintained on a standard 12:12 light cycle (lights on 0700-1900h, lights off 1900-0700h). Experiments to manipulate mu-opioid receptor-expressing neurons used MOR-Cre mice developed in our lab [28]. Briefly, the MOR-specific Cre mouse line was generated by inserting a T2A cleavable peptide sequence and the Cre coding sequence into the Oprm1 3’UTR. Importantly, this line shows specificity and fidelity of MOR expression throughout the brain and no differences in behavioral responses to morphine when compared to wild-type mice. These MOR-Cre mice were then crossed with *Rosa*^*LSLSun1-sfGFP*^ [29] to generate a reporter line for visualization of MORs or *Rosa26*^*LSL-EGFP-L10a*^ [30] for cell-type specific RNA sequencing. Mice were 8–12 weeks at the start of each experiment.

### Drugs and experimental design

Morphine sulfate was supplied by NIDA Drug Supply (Research Triangle Park, NC) and dissolved into a 0.2% saccharin solution so that mice would consume morphine via drinking water [31]. Saccharin was used in the baseline condition and in the control groups for the duration of the studies. Morphine concentration was gradually increased from 0.3 to 0.5 mg/ml to ensure stable drinking throughout the 11 days of chronic exposure. Dosing stayed at 0.5 mg/ml for the remainder of the experiments [32]. Body weights and drinking volume were assessed every 2–3 days to confirm morphine ingestion, and adequate body weight was maintained. Clozapine N-Oxide (CNO) (HelloBio, cat. no. 1807) was dissolved in saline and administered i.p. at 1 mg/kg.

### Electroencephalogram/electromyogram (EEG/EMG)

**Fig. 1:**
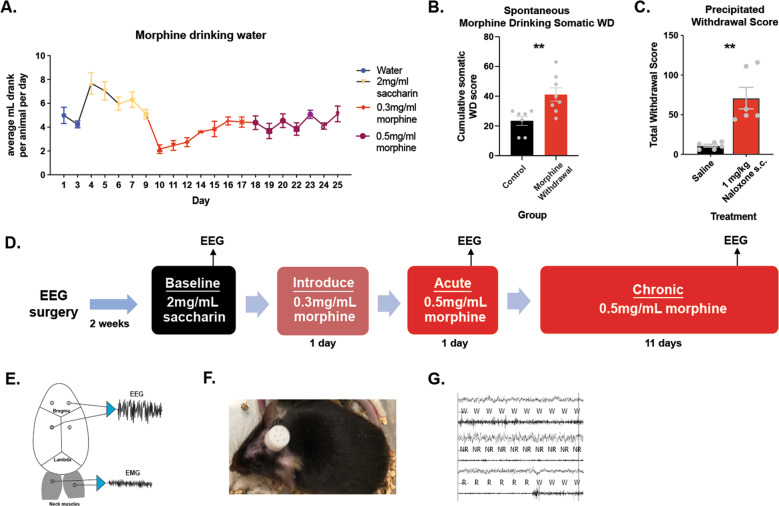
Experimental Design. **A** Amount consumed in drinking water in average mL per animal per day. **B** Spontaneous somatic withdrawal signs between morphine-treated mice and saccharin-treated mice. **C** Somatic withdrawal signs in mice that drank morphine precipitated by 1 mg/kg Naloxone or Saline. **D** Timeline of experiments. **E** Example schematic showing placement of electrodes 1 mm from bregma in each quadrant and on the nuchal muscles with example EEG and EMG traces. **F** Image of mouse with electrode cap. **G** Example traces of Wake, NREM, and REM in 4 s epochs.

Mice were anesthetized with a ketamine/xylazine cocktail for surgical implantation of electrodes. Four holes were drilled 1 mm from bregma in each quadrant. A headmount was secured with silver wire coated in Teflon (787000, A-M Systems, Sequim, WA) and pedestals with six pins (MS363, PlasticsOne). Dental cement (8101 and 8501, Pearson Dental, Sylmar, CA) secured these to the head (Fig. 1E, F). EMG electrodes were placed over the nuchal muscles and connected to a computer with a counterweighted recording cable. Mice recovered for 2 weeks before starting EEG experiments and acclimated to the EEG room for 1 week before experiments. Raw files were opened in Sleep Sign for Animal (Kissei) after being amplified, recorded, and digitized. Wake, NREM, or REM was categorized in 4 s epochs according to the following conditions: wake-waveforms greater than 12 Hz greater than 10% of the time with high amplitude EMG. NREM- 0.5–4 Hz delta waves in the EEG more than 30% of the time and frequencies greater than 12 Hz less than 10% of the time with low amplitude EMG. REM- 5–10 Hz EEG delta waves more than 20% of the time with low EMG amplitude (Fig. 1G) [33]. After digitization, each 4 s epoch was manually verified and rescored by a trained experimenter if necessary.

### Continuous open mouse phenotyping and sleep system (COMPASS)

Sleep as measured by behavioral quiescence was monitored noninvasively as described previously [34, 35]. This COMPASS system uses passive infrared sensors which are attached to the cage top to monitor and document activity continuously via an open-source electronics platform which records active movement by receiving inputs from multiple sensors (Arduino). Activity is collected and the software generates a score from 0 to 100 based on 10 ms recordings for a bin every 10 s, denoting the percent of time mice were moving in that 10 s bin. Extended inactivity (0 score) greater than 40 s correlates with sleep measured by electroencephalography with a Pearson coefficient of greater than 0.95 [35]. We used a custom program in Python to convert 40 s of inactivity into sleep and to identify bouts of sleep that lasted longer than 40 s.

### Somatic signs of withdrawal

Precipitated withdrawal was examined immediately after a 1 mg/kg subcutaneous (s.c.) dose of naloxone, or equivalent volume of saline for controls, and spontaneous withdrawal was conducted 24 h after morphine was removed. Mice were acclimated in the behavior room 1 h prior to observations and were placed in a clear cylinder for 30 m before testing began. Somatic withdrawal signs were scored live and the total number of episodes of ptosis, diarrhea, teeth chattering, resting tremor, gnawing, genital licking, head and body shakes, backing, scratching, and jumping were tallied as described previously [32, 36].

### Viruses and stereotaxic surgery

The Cre-recombinase dependent inhibitory DREADDs virus was from Bryan Roth, pAAV5-hSyn-DIO-hM4D(Gi)-mCherry (cat. no. 44362, Addgene). The control virus was from Karl Deisseroth, pAAV5-hSyn-mCherry (cat. no. 114472, Addgene). Mice were anesthetized with isoflurane and placed in a stereotax to deliver 200 nl virus into the PVT at a rate of 40 nl/min at coordinates A/P—1.58 mm, D/V—3.20 mm [37]. Mice received subcutaneous meloxicam (2 mg/kg) on the day of surgery. For optimal virus expression, the virus was allowed to express for 4 weeks before behavioral studies or brain collection.

### Translating ribosome affinity purification (TRAP)

We used both control mice and mice exposed to chronic morphine for TRAP. Mice were sacrificed between 1200 and 1400 h and, for each mouse, a gross dissection targeted at the PVT was used to generate tissue for the translating ribosome affinity purification. Due to the small amount of brain tissue, 2 PVT dissections were pooled together to create each biological sample—males were only pooled with males and females were only pooled with females, for a total of four biological replicates for each of the morphine and control groups. Tissue was homogenized with lysis buffer and incubated overnight with magnetic beads (Dynabeads Protein G, ThermoFisher Scientific, Waltham, MA, cat. no. 10004D) and anti-GFP antibodies (htz-GFP-19f and htz-GFP-19C8, Memorial Sloan-Kettering Monoclonal Antibody Facility, New York, NY) to complete the affinity purification bound by the EGFP-L10a fusion protein. RNA was isolated from the cells in each sample expressing EGFP-L10a [30, 38] with the Absolutely RNA Nanoprep RNA isolation kit (Agilent Technologies, cat. no. 400753).

### RNA sequencing

RNA was processed, assessed for quality control, and prepared and sequenced by the University of Pennsylvania’s Next Generation Sequencing Core (https://ngsc.med.upenn.edu). Briefly, libraries were sequenced as 50PE and samples had between 37 and 58 million reads. We quantified transcript expression with Salmon [39] in mapping-based mode with the mm10 salmon_sa index obtained from http://refgenomes.databio.org and GC bias correction. Differential Expression (DE) analyses were done with DESeq2 [40]. We followed the DESeq2 recommended pipeline to create a gene-level count matrix by importing the Salmon quantification data using tximport [41]. Genes with 0 counts across all samples were filtered out. (22,402 out of the ~35,650 gene models passed this filter). Exploratory QC plots did not reveal a strong sex effect on the gene expression data. Moreover, after analyzing the data both with and without sex in the design model, we obtained very similar DE results with slightly more genes without the adjustment. Therefore, in what follows, we report the results from the latter analyses. For enrichment analyses, we focused on the KEGG_2019_Mouse pathway collection from https://maayanlab.cloud/Enrichr/#libraries. We ran Gene Set Enrichment Analysis (GSEA) [42] and Over Representation Analysis (ORA, implemented via the enricher function in the R clusterProfiler package [43]. EnhancedVolcano was used to generate the volcano plot [44]. In both cases we only considered gene sets with a number of genes between 15 and 500. GSEA (v4.2.3) was run with default parameter settings except for “permutation_type” which we set to “gene_set”, as this is the recommended setting when the number of replicates per condition is below 7.

### Immunohistochemistry

Mice were deeply anesthetized with sodium pentobarbital (i.p., 50 mg/kg) and were perfused with 30–40 ml cold 0.01 M phosphate-buffered saline (PBS), then 40–50 ml cold 4% paraformaldehyde (PFA) (Sigma, cat. no. 158127). Perfusions for this study took place between 1000 and 1400 h. Brains were placed in a 15 ml conical tube with paraformaldehyde overnight and cryoprotected 24 h later in 30% sucrose and 0.01% sodium azide and stored at 4 °C. Brains were then sliced frozen at −20 °C on a cryostat (Cryostar NX-50, Thermo Scientific, Waltham, MA), and 30-micron sections were collected and stored in PBS in a six-well plate at 4 °C. After mounting sections onto slides (Superfrost Plus, ThermoFisher, cat. no. 22-037-246, Waltham, MA), sections were washed 4 × 10 m in 0.01 M PBS, blocked for 1 h in 0.01 M PBS containing 3% normal Normal Donkey Serum (NDS) and 0.3% Triton X-100, and incubated overnight at 4 °C with a 1:300 dilution of rabbit anti-cFOS (Cell Signaling Technologies, cat. no. 2250 S). Sections were again washed in 0.01 M PBS and incubated with Alexa Fluor 488 donkey anti-rabbit (1:1000, Life Technologies, cat. no. A-21206) in 3% NDS in PBS for 1 h at room temperature while protected from light. Slides were coverslipped with DAPI Fluoromount (SouthernBiotech, cat. no. 0100-20, Birmingham, AL) and sealed with clear nail polish prior to imaging.

### Statistics

Statistics were analyzed and plotted in GraphPad 8.0 (La Jolla, CA). Unpaired *T* tests were used to compare between two groups. One-way repeated measures ANOVAs were used to compare within subjects at different timepoints compared to their own baseline. Two-way ANOVAs were used for tests with two independent variables, and Tukey’s post hoc test was employed when there was a main effect or interaction effect from the ANOVA. A three-way ANOVA was used to compare DREADD × morphine × day, and Tukey’s post hoc test was used when a main or interaction effect was significant.

## Results

Mice exposed to morphine drank about 5 mL per animal per day (Fig. 1A). At a dose of 0.5 mg/ml, this amounts to approximately 100 mg/kg morphine per animal per day for mice at a 25 g weight. This exposure paradigm was sufficient to generate dependence on morphine, as morphine-treated mice displayed significantly increased signs of either spontaneous somatic withdrawal compared to mice that did not receive morphine (*t* = 2.164, df = 13, *P* = 0.0075) (Fig. 1B), or precipitated withdrawal compared to mice that received saline instead of Naloxone (*t* = 4.386, df = 10, *P* = 0.0014) (Fig. 1C).

**Fig. 2:**
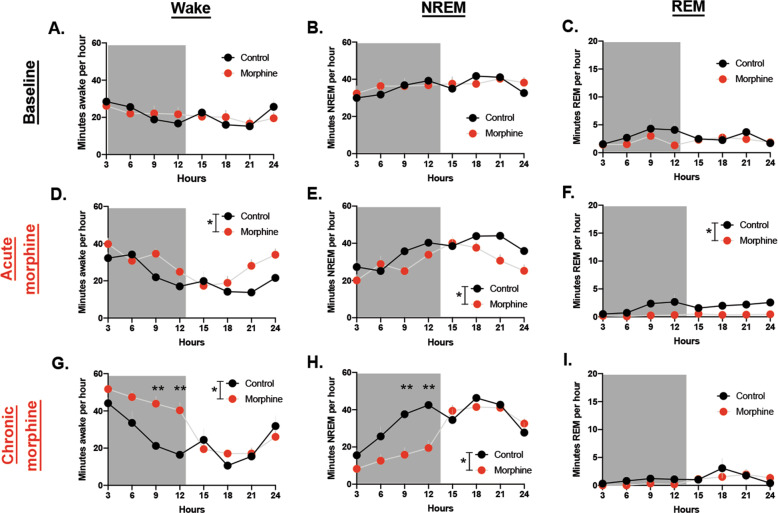
Morphine increases wake during the dark cycle. **A** Minutes awake per hour at baseline. **B** Minutes in NREM per hour at baseline. **C** Minutes REM per hour at baseline. **D** Minutes awake per hour at acute morphine timepoint. **E** Minutes NREM per hour at acute morphine timepoint. **F** Minutes REM per hour at acute morphine timepoint. **G** Minutes awake per hour at chronic morphine timepoint. **H** Minutes NREM per hour at chronic morphine timepoint. **I** Minutes REM per hour at chronic morphine timepoint.

At baseline, there was no difference between control and morphine groups (before morphine was administered) in time awake (*F* (1, 10) = 5.473, *P* = 0.9942) (Fig. 2A). There were no significant differences in NREM (*F* (1, 10) = 0.02383, *P* = 0.8804) (Fig. 2B) or REM sleep (*F* (1, 10)—0.8026, *P* = 0.3914) at baseline (Fig. 2C).

At the acute morphine timepoint, there was a main effect of morphine on wake (*F* (1, 8) = 7.48, *P* = 0.0256) (Fig. 2D). There was no significant interaction effect of morphine × hour (*F* (7, 56) = 1.959), *P* = 0.0772) (Fig. 2D), nor significant post hoc tests at any specific timepoint. There was also a main effect of acute morphine on NREM sleep (*F* (1, 8) = 6.842, *P* = 0.0309) (Fig. 2E) and no interaction effect (*F* (7, 56) = 1.810, *P* = 0.1033) nor specific post hoc tests that were significant. At the acute timepoint there was a significant effect of morphine on REM (*F* (1, 8) = 15.00, *P* = 0.0047) and a significant interaction effect of morphine × hour (*F* (7, 56) = 4.932, *P* = 0.0002) (Fig. 2F).

At the chronic morphine timepoint, there was a significant main effect of morphine (*F* (1, 11) = 6.255, *P* = 0.0295) (Fig. 2G) on time awake and a significant interaction effect of morphine × hour (*F* (7, 77) = 4.356, *P* = 0.0004) (Fig. 2G). Post hoc tests revealed a significant difference between the control and morphine group specifically in the last 6 h of wake in the dark cycle (*P* = 0.0041 and *P* = 0.0014) (Fig. 2G). There was also a main effect of morphine on NREM at the dark cycle (*F* (1, 11) = 5.684, *P* = 0.0362) (Fig. 2H) and a significant interaction effect of morphine × hour (*F* (7, 77) = 4.539, *P* = 0.0003) (Fig. 2H). Post hoc tests revealed a significant difference in NREM between control and morphine in the last 6 h of the light cycle (*P* = 0.0019 and *P* = 0.0016) (Fig. 2H). There was no significant effect of chronic morphine on REM sleep. Morphine increased wakefulness for 3–4 h during the light cycle in an injection paradigm, confirming morphine’s wakefulness properties in a different paradigm (Supplementary Fig. 1a).

**Fig. 3:**
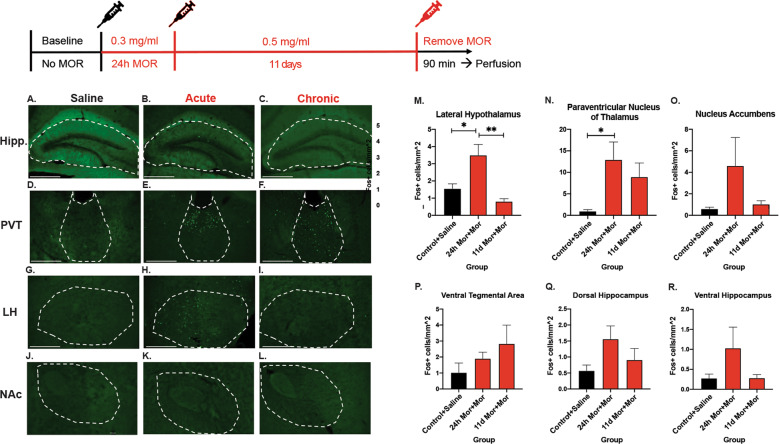
Screen of immediate early gene expression across brain regions. **A** Representative image of hippocampus from saline group. **B** Hippocampus from acute morphine group. **C** Chronic morphine hippocampus. **D** Representative image of PVT (paraventricular nucleus of the thalamus) from saline group. **E** PVT from acute morphine group. **F** Chronic morphine PVT. **G** Representative image of LH (lateral hypothalamus) from saline group. **H** LH from acute morphine group. **I** Chronic morphine LH. **J** Representative image of NAc (nucleus accumbens) from saline group. **K** NAc from acute morphine group. **L** Chronic morphine LH. **M** Quantification of cFOS expression in LH across morphine timepoints. **N** cFOS expression in PVT across morphine timepoints. **O** cFOS expression in NAc quantified across morphine timepoints. **P** Quantification of cFOS expression in VTA across morphine timepoints. **Q** cFOS expression levels in Dorsal Hippocampus across morphine timepoints. **R** cFOS expression in ventral hippocampus at control, acute, and chronic morphine. Images were taken at 10× quantification and scale bars represent 500 um.

We examined protein levels for the immediate early gene cFos in a number of brain regions that are related to opioid reward, response, or memory. In the lateral hypothalamus, the acute timepoint had significantly increased cFOS expression compared to control and chronic morphine (*F* (4, 22) = 5.927, *P* = 0.022) (Fig. 3M) and post hoc tests revealed acute morphine was significantly higher than baseline (*P* = 0.0355) and chronic morphine (*P* = 0.011). In the Paraventricular Nucleus of the Thalamus (PVT), cFOS was significantly higher than baseline at the acute timepoint (*F* (2, 16) = 3.224, *P* = 0.0327), and the chronic morphine timepoint was not significantly different from the acute timepoint (*P* = 0.6598) (Fig. 3N). None of the other brain regions (nucleus accumbens (*F* (2, 16) = 1.625, *P* = 0.2345), ventral tegmental area (*F* (2, 16) = 0.9067, *P* = 0.4718), dorsal (*F* (2, 17) = 1.849, *P* = 0.1877) or ventral hippocampus (*F* (2, 16) = 1.409, *P* = 0.2821) reached statistical significance (Fig. 3O–R).

**Fig. 4:**
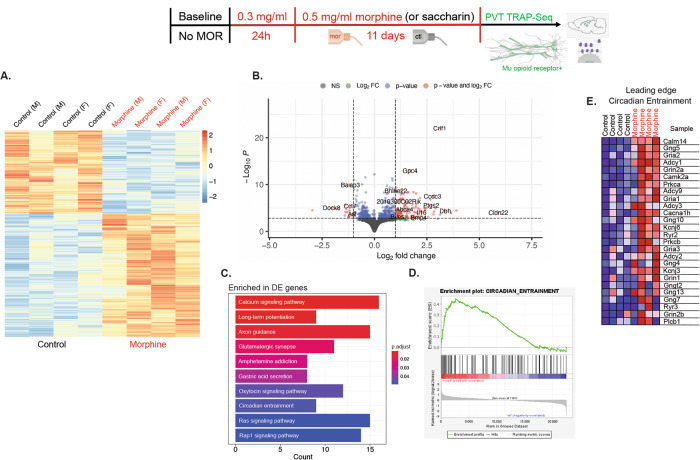
Translating ribosome affinity purification on MOR-expressing neurons targeted at the paraventricular nucleus of the thalamus. **A** Heatmap of significantly differentially expressed genes in PVT MOR-expressing neurons of controls compared to mice maintained on chronic morphine. **B** Volcano plot showing −log10*P* value vs log2 fold change (morphine vs ctl group) for all genes analyzed. Genes corresponding to significant adjusted *P* values are those above the dashed horizontal line. Dashed vertical lines correspond to a threshold of 1 for the absolute log2 fold change. **C** Significant KEGG pathways from ORA analyses of DE genes. **D** Enrichment plot of the Circadian Entrainment pathway. **E** Heatmap of the genes in the leading edge of the Circadian Entrainment pathway.

RNA sequencing of the MOR + expressing neurons in the PVT that were affinity purified by Translating Ribosome Affinity Purification (TRAP) and subsequent DE analysis identified 311 upregulated and 211 downregulated genes in the morphine group as compared to the control group (multiple-testing-adjusted *P* value <0.05. The list of these genes, complete with the DESeq2 output is in Supplementary Table 1. Figure 4A depicts a heatmap of the DE genes and Fig. 4B presents a volcano plot of all genes analyzed in DESeq2. Of the KEGG gene set collection, 285 pathways out of 303 had a number of genes between 15 and 500 and were analyzed in our ORA and GSEA analyses. Figure 4C illustrates the ten pathways that were significantly enriched among all 522 DE genes (multiple-testing adjusted *P* values <0.05). These include the Circadian Entrainment and Glutamatergic Synapse pathways, which were also overrepresented among just the 311 upregulated genes in the morphine group (data not shown). ORA analyses focus on specific lists of genes (e.g., the DE genes) and look for gene sets which are overrepresented in those lists compared to the universe of all genes examined. GSEA analyses instead have gene sets as the starting point and look for gene sets that, as a whole, have a trend towards up- or downregulation, whether or not the individual genes in the gene set are per se differentially expressed at a given significance level. The KEGG pathways identified in our GSEA analyses with a false discovery rate <5% are indicated in Supplementary Table 2A, B. We note that the Circadian Entrainment and Glutamatergic Synapse pathways are detected for a trend of upregulation in the morphine group, consistent with the ORA results. Figure 4D shows the GSEA enrichment plot for KEGG Circadian Entrainment and (Fig. 4E) provides an expression gene map for the genes in its leading edge, i.e., the genes which contributed the most to the enrichment signal for this pathway.

**Fig. 5:**
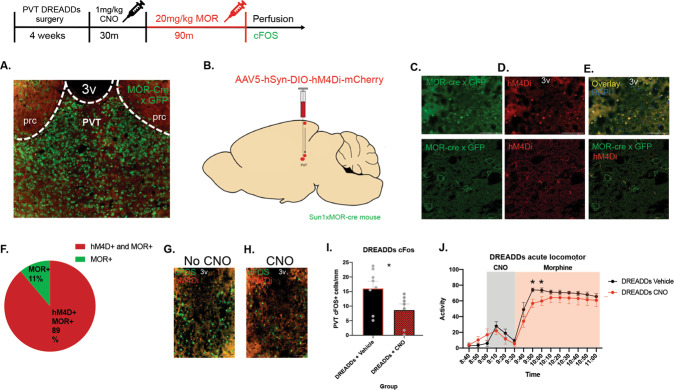
DREADD inhibition of neurons in the PVT that express mu-opioid receptors. **A** Representative image of mu-opioid receptors in the PVT with MOR Cre x GFP reporter mouse. **B** Virus strategy to target PVT MOR-expressing neurons. A Cre-dependent inhibitory DREADDs construct was injected into the PVT of mice that express Cre recombinase on the nucleus of mu-opioid receptor-expressing neurons. **C** Mu-opioid receptor-expressing neurons identified by GFP reporter in the PVT at 10× and super-resolution. **D** hM4Di expression in the PVT identified by mCherry reporter at 10× and super-resolution. **E** Overlay of MOR-expressing neurons in green and DREADDs construct in red at 10× and super-resolution. **F** quantification of transfection of MOR + neurons in the PVT that also express hM4Di. **G** Representative image of cFOS expression in the PVT of mice injected with the DREADDs virus. **H** Representative image of cFOS expression in the PVT of mice injected with the DREADDs virus after i.p. CNO injection. **I** Quantification of cFOS expression in the PVT to show DREADD inhibition from CNO. **J** Activity plot per 10 m bins of mice that received CNO or saline injection with a morphine stimulus. Scale bars represent 100 μm.

In the DREADDs injected mice, 89% of neurons that expressed MORs also expressed mCherry from the DREADDs construct (Fig. 5E, F). CNO administration showed significantly decreased PVT cFOS expression in the DREADDs CNO group compared to the DREADDs vehicle group (*t* = 2.270, df = 13, *P* = 0.0409) (Fig. 5G–I), and in a locomotor activity assay, the DREADDs + CNO group had significantly decreased morphine-induced activity 20 and 30 min after morphine injection compared to the DREADDs + saline group (main effect of time *F* (14, 84) = 76.60, *P* < 0.0001 (Fig. 5J).

**Fig. 6:**
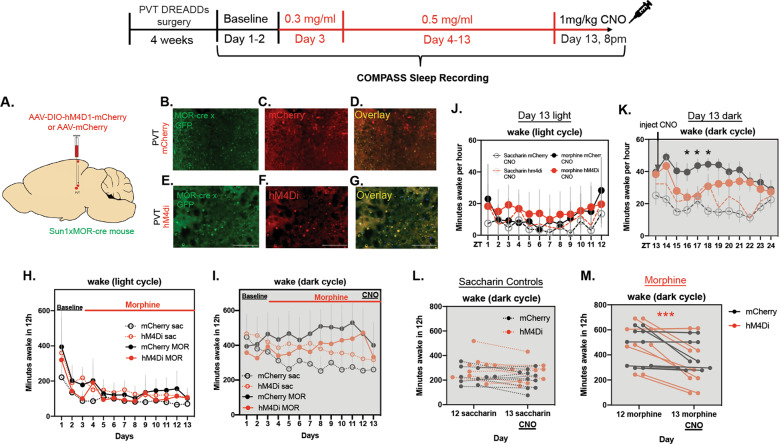
DREADDs inhibition of PVT MOR-expressing neurons during chronic morphine. **A** Representative image showing viral strategy- Cre-dependent DREADDs virus or control mCherry virus was injected into the PVT of MOR-Cre mice. **B** Representative image of MOR expression in the PVT in mCherry animals. **C** mCherry expression in the PVT. **D** Overlay of mCherry and MOR expression. **E** Representative image of MOR expression in the PVT in DREADDs injected mice. **F** Representative hM4Di expression in the PVT. **G** Overlay of PVT MOR and hM4Di expression. **H** Minutes awake per 12 h during the light cycle. **I** Minutes awake per 12 h during the dark cycle. CNO was injected 1 h into the dark cycle on the last day (13) of morphine treatment. **J** Minutes awake per hour in the light cycle immediately before CNO administration. **K** Minutes awake per hour in the dark cycle, including CNO administration 1 h into the dark period at 8 pm. **L** Minutes awake per 12 h in the dark cycle preceding CNO administration and the dark cycle including CNO administration in mice that did not receive morphine. **M** Minutes awake per 12 h in the dark cycle preceding CNO administration and the dark cycle including CNO administration in mice that were treated with morphine. Scale bars represent 100 μm.

In the DREADDs chronic morphine experiment, all groups had similar wake times for the first few days, and mice in the morphine group began to separate with increased wake time in the dark cycle compared to the saccharin group by days 11–12 (Fig. 6I). There was a morphine × DREADDs interaction effect during the dark cycle (*F* (1, 25) = 5.357, *P* = 0.0291) (Fig. 6I). Much of the sleep patterns were similar between groups on the days before CNO administration (Supplementary Fig. 2A–C). There were no differences in sleep bouts—number, average length, or maximum (*F* (3,35 = 1.935, *P* = 0.1419)) on days 1–12 (Supplementary Fig. 2A–C). However, CNO injection after the chronic morphine exposure produced a longer average sleep bout length in the hM4Di morphine group compared with the mCherry morphine group (*t* = 1.720, df = 17, *P* = 0.0418) (Supplementary Fig. 2E). The sleep bout # and maximum sleep bout length were unchanged between these two groups (Bout # *t* = 0.04409, df = 17, *P* = 0.9653), (bout length *t* = 1.179, df = 17, *P* = 0.2547) (Supplementary Fig. 2d, f).

PVT MOR neuron inhibition during chronic morphine produced no wake differences between groups in the light cycle immediately preceding CNO administration (*F* (11, 339) = 0.9187, *P* = 0.5224) (Fig. 6J). In the dark cycle, when CNO was given at 8 pm, 1 h after the start of lights off, CNO decreased wakefulness only in the morphine hM4di group compared to the morphine mCherry group (*F* (11, 385) = 2.225, *P* = 0.0127) (Fig. 6K). This significant effect was between 10 pm and 1 am, beginning 2 h after CNO injection and ending 5 h after CNO injection. There was a main effect of morphine to increase wakefulness, confirming earlier results (*F* (1, 35) = 4.908, *P* = 0.0333) (Fig. 6K). In the full 12-h period of the dark cycle on the last day before CNO, there was no significant difference in wake time between hM4D and mCherry, for either the morphine group or the saccharin controls (*F* (1,17) = 0.06110, *P* = 0.8077) (Fig. 6L, M). However, on the day CNO was administered, there was a significant decrease in dark cycle wake time after CNO compared to the previous dark cycle, only in the morphine DREADDs group (main effect of CNO, (*F* (1, 17) = 20.33, *P* = 0.0003, and main effect of subject (*F* (17, 17) = 7.076, *P* = 0.001) (Fig. 6M).

## Discussion

Here, we provide evidence that acute morphine exposure increases wakefulness in a non-circadian manner, and in contrast, chronic morphine exposure disrupts sleep in mice in a circadian-specific (dark cycle) manner and that this increased wakefulness requires activation of the MOR + PVT neurons. Remarkably, wake during the light cycle was relatively undisturbed. Animals randomized to the morphine arm of our paradigm displayed both spontaneous and precipitated somatic signs of withdrawal, indicating the mice were dependent on morphine at the time of the prominent sleep disturbance.

Animals self-administered approximately 100 mg/kg of morphine per day. While 100 mg/kg is a high dose of morphine, this dose is distributed over 24 h. Due to the morphine in the drinking water, animals necessarily had to be awake and actively accessing the drinking bottle to acquire morphine. This generates the possibility that mice were only staying awake to access the rewarding substance, not that morphine itself caused wakefulness. However, the wakefulness was specific to the second half of the lights-off period and no effect was observed for the 12-h lights on period. Moreover, our escalating dose injection paradigm also demonstrated biological (not motivational) wakefulness properties of morphine (~3–4 h), though it was not as sustained as in the drinking paradigm (the entire dark cycle, ~12 h). In the injection paradigm, the mice increase wakefulness as long as the morphine is present in their system. Overall, morphine at this dose has both wake-generating properties and increases motivation to stay awake, at least throughout the dark cycle.

C-Fos is expressed within neurons following depolarization and is therefore often used as a marker for neuronal activity. Analysis of cFOS protein expression revealed that neurons in the paraventricular nucleus of the thalamus maintained elevated cFOS levels at the end of the oral morphine self-administration paradigm compared to other reward-related brain regions, suggesting sustained neuronal activity in this region. Thus, we focused on the PVT as a likely site of mechanism underlying our sleep phenotype. Using a mouse that expresses Cre recombinase in mu-opioid receptor-expressing neurons, we employed TRAP to characterize the transcriptome of these MOR-expressing neurons in the PVT. Sequencing of MOR neurons in the PVT at the chronic morphine timepoint highlighted the circadian entrainment pathway as significantly upregulated in the morphine group. We then sought to manipulate these PVT MOR-expressing neurons to see whether they are necessary for morphine-induced changes in the sleep-wake cycle. Conditionally inhibiting these PVT MOR + neurons blocked the morphine-specific wakefulness for the expected duration of CNO effect (30 m–4 h) [45], therefore, only when CNO is active is there a wake effect. It is possible that the COMPASS system was not sensitive enough to capture the CNO effects in the first two hours or that neuronal inhibition does not instantly decrease wakefulness, and mice do not wake up immediately after the CNO wears off.

With the PVT as a locus of interest relating to chronic morphine and wakefulness, we profiled only Mu Opioid Receptor-expressing neurons in the PVT in morphine compared with controls. The overall effect was more upregulated genes in response to morphine compared to significantly downregulated genes, yet little difference in gene expression patterns between males and females (Fig. 4A). Only ten biological pathways were significantly overrepresented among the genes that were significantly different between controls and mice exposed to morphine (Fig. 4C). Among those, and also among the significantly upregulated pathways (in the morphine group) in the GSEA analysis, was the Circadian Entrainment pathway (Fig. 4D). This pathway contains many genes related to the biological clock and synchronizing circadian and behavioral rhythms to environmental cues such as lights on or lights off (Fig. 4E). Four (*Adcy* 1, 2, 3, 9) of the nine subtypes of adenylate cyclase are on the GSEA leading edge (i.e., the genes that contributed significantly to the enrichment signal) for Circadian Entrainment (Fig. 4E). Adenylate cyclases play important roles in diverse brain functions including synaptic plasticity, and show strong to medium expression in the thalamus and striatum [46]; brain regions that are part of both sleep/wake and reward circuitry. Adcy1 is one of two adenylyl cyclases that are activated by increases in Ca2 + , which is the top enriched pathway upregulated in morphine-exposed mice. As adenylate cyclases are critical in neuronal signaling, perturbation of their activity by opioid exposure could influence any number of processes critical for sleep/wake and reward-related behaviors.

Further, one of the top significantly upregulated genes in morphine-exposed mice was Dopamine Beta Hydroxylase (Dbh) (Fig. 4B). DBH is the enzyme that converts dopamine to norepinephrine, and is likely to have an influence on sleep through its role in norepinephrine production [47]. Norepinephrine-containing neurons in the locus coeruleus project throughout the brain and their activation contributes to attention, arousal, and autonomic activation to support these processes. Activity of locus coeruleus neurons is maximal in wakefulness. Mice lacking Dbh have altered sleep latency after mild stress and administration of amphetamine [48]. CRISPR-induced knockdown of Dbh in the locus coeruleus (LC) reduces wakefulness when the LC is optogenetically stimulated [49]. While our tissues were collected from the PVT not the LC, it is still possible that, based on these results, Dbh in the PVT may contribute to drug-induced sleep/wake alterations. Other genes from the leading edge of the Circadian Entrainment pathway that may have a role in the morphine-sleep relationship include Grin2a [50], Camk2a [51], Prkca [52], and Gria1 [53] (Fig. 4E). Of interest, these genes are all either directly activated by calcium (Prkca, Camk2a) or regulate calcium response (Grin2a, Gria1) indicating an important role for this signaling molecule in mediating wake regulation following opioid exposure. It is important to note that in the TRAP-Seq experiment, sleep was not manipulated; the only independent variable was morphine administration, yet Circadian Entrainment is one of the few overrepresented pathways among DE genes. This result lends credence to the hypothesis that sleep and opioids are fundamentally and biologically intertwined [1].

Our study only manipulated the PVT MOR + expressing neurons, not their inputs or projections. Future circuit tracing studies could identify the inputs and outputs of MOR PVT neurons, and manipulating each part of the circuit could identify the critical neurons or circuits for controlling sleep in the PVT. The PVT is a major source of afferents to the nucleus accumbens which itself is a critical nucleus for wakefulness as well as being part of the mesolimbic dopamine reward pathway [54]. However, we did not see a significant increase in cFOS expression in the nucleus accumbens. This may be due to the fact that the nucleus accumbens also contains sleep-promoting cells [55], which may have prevented any further increases in cFOS expression in this region.

The morphine-specific sleep/wake phenotype was evident during the dark cycle but not the light cycle. Given the time-dependent circadian effect on wakefulness via opioid receptors in the PVT [23], orexin neurons may be one possible input contributing to this phenotype. Orexins are synthesized in the lateral hypothalamus (LH) and project widely throughout the brain, including to the PVT, which contains orexin 1 receptors and orexin 2 receptors [56–58]. These orexinergic projections from the LH to PVT mediate sleep/wake behaviors in the absence of drugs [24]. The involvement of hypothalamic orexin neurons in the PVT with respect to drug use has been a recent area of research [59], however the specific role and the synaptic physiology of orexin neurons and mu opioid receptors in the PVT is not fully understood and should be investigated in both non-drug and drug conditions.

The mu opioid receptor is an inhibitory, Gi-coupled receptor, and we expressed an inhibitory, Gi-coupled DREADDs construct in cells that expressed Cre recombinase driven by the mu-opioid receptor. DREADD inhibition of MOR + PVT neurons could have created more inhibition than morphine typically provides. Although morphine increases wakefulness in experimental settings, it is a sedative at high doses, and it is possible our DREADD inhibition of MOR neurons forced these mice into a more sedated state, similar to high doses of morphine. Morphine increases tonic firing and initial burst firing in the PVT, at least in the light cycle [23]. It will be important to consider more system-wide, circuit-based experiments to determine how MOR PVT neurons connect and affect signaling in the PVT and throughout the rest of the brain. Of note, among the enriched pathways significantly upregulated in the morphine group in both the ORA and GSEA analyses were Glutamatergic Synapse (see Supplementary Fig. 3), and Calcium Signaling, indicating that MOR PVT neurons intricately affect excitatory circuitry, and potentially morphine creates long-lasting changes in these neuronal connections.

In the DREADDs experiment, we used the COMPASS system instead of EEG sleep recording. We did this in order to power the study with enough animals, as the EEG system and analysis limits the number of animals tested at any one time, while the COMPASS system allows for large-scale analysis and served to confirm the phenotype in a different system. The COMPASS system may overestimate sleep, as any period of extended locomotor inactivity is counted as sleep [34, 60]. However, we did see the same general wake pattern between EEG and COMPASS where morphine increased wakefulness during the dark cycle but had little impact during the light cycle (Fig. 6H, I). We did not examine spectral power in this study to determine whether the wake state at baseline is different than the wake state during chronic morphine, which is a limitation as spectral power can be affected by sleep deprivation [61]. The COMPASS study shows PVT MOR + neurons mediate chronic morphine-induced wake properties, but EEG studies would be needed to examine the full effect of these neurons on morphine-induced sleep.

Interestingly, DREADD inhibition of MOR + cells in the PVT blocked wakefulness only in the morphine group, not in the control group. To our knowledge, this is the first manipulation that changed drug-induced wakefulness without affecting general wakefulness (Fig. 6L, M). However, one study has shown that MORs contribute to morphine-induced wakefulness in rats, as morphine hyperpolarizes sleep-promoting neurons in the Ventrolateral Preoptic Area (VLPO), and is blocked by the MOR antagonist CTOP [20]. It is possible that there was only decreased wakefulness in the morphine DREADDs group due to a floor effect. As morphine increases wakefulness during the dark cycle (Figs. 2 and 3), there is more room to decrease wakefulness in the morphine DREADDs group (~500 m awake) compared to the control (saccharin) DREADDs group (~250 m awake).

Overall, this is one of the first studies to examine the effects of chronic morphine on sleep and the first of our knowledge to identify neurons that, when manipulated, alter drug-induced wakefulness without affecting general wakefulness. That chronic morphine exposure increases wakefulness during the dark cycle was confirmed with both EEG and COMPASS analysis, and morphine transiently increased wakefulness during the light cycle in an injection paradigm. Future experiments could manipulate sleep pharmacologically and assess opioid reward. This research should be taken in context with the clinical literature showing sleep disturbance in OUD patients; it may be possible to treat sleep in order to ameliorate the negative symptoms associated with OUD and withdrawal. Indeed, clinical trials have begun to test FDA-approved drugs for insomnia with a possible outcome of decreasing rates of relapse in OUD (NCT04262193). At the least, sleep should be an important consideration in the overall treatment of not just addiction but other neuropsychiatric disorders.

## Supplementary information

Supplementary Figure Legends

SFigure 1

SFigure 2

SFigure 3

Supplementary Table 1

Supplementary Table 2

## Data Availability

Our TRAP-Seq data are available with GEO accession number GSE214560.
