# Supplementary material for: Mu-opioid receptor-expressing neurons in the paraventricular thalamus modulate chronic morphine-induced wake alterations
Source: Transl Psychiatry. 2023 Mar 3;13:78. doi: 10.1038/s41398-023-02382-w (PMC9984393; doi:10.1038/s41398-023-02382-w)
Supplement: Supplementary file 1 — Supplementary Figure Legends [file 41398_2023_2382_MOESM1_ESM.docx]

**Supplementary Figure 1.** Morphine effects of wakefulness with injection instead of oral administration, measured by EEG. A) Minutes awake per hour in an escalating morphine dose paradigm where mice were injected subcutaneously with morphine twice per light cycle at increasing doses. B) Minutes awake per 12h during the escalating dose morphine injection paradigm for both the dark cycle and light cycle. C) Minutes NREM per 12h during the escalating dose morphine injection paradigm for both the dark cycle and light cycle.

**Supplementary Figure 2.** Sleep bout patterns across day 1-12 and on day 13. A) Average number of bouts per day on day 1-12. B) Average length of each sleep bout on day 1-12. C) Maximum sleep bout length on day 1-12. D) # of bouts on Day 13, the day CNO was administered. E) Average length of sleep bouts on Day 13, the day CNO was administered. F) Maximum bout length on Day 13, the day CNO was administered.

**Supplementary Figure 3.** Enrichment plot of the Glutamatergic Synapse pathway.
