# Supplementary figures and images for: Mu-opioid receptor-expressing neurons in the paraventricular thalamus modulate chronic morphine-induced wake alterations
Source: Transl Psychiatry. 2023 Mar 3;13:78. doi: 10.1038/s41398-023-02382-w (PMC9984393; doi:10.1038/s41398-023-02382-w)

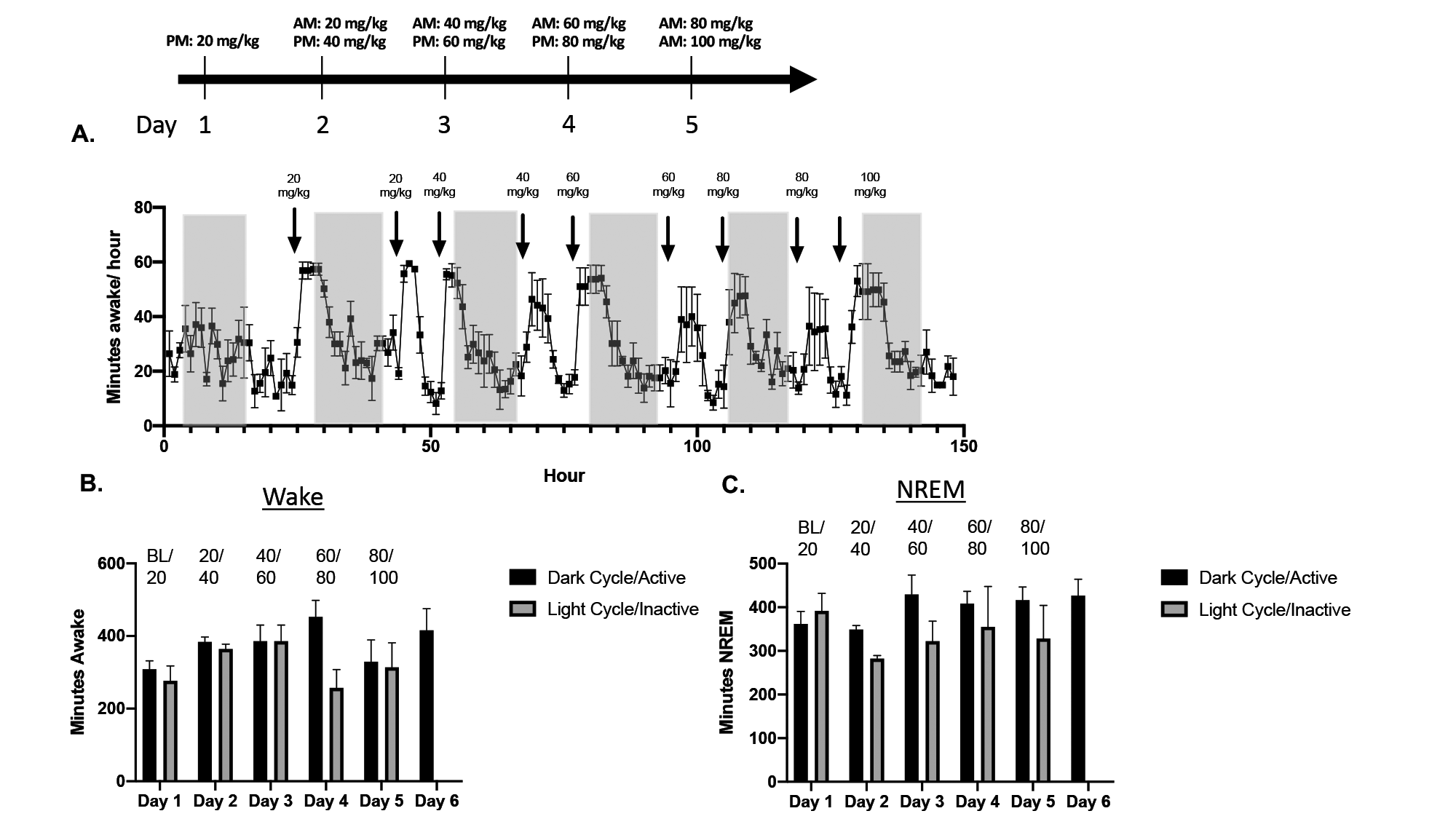

Supplement: Supplementary file 2 — SFigure 1 [file 41398_2023_2382_MOESM2_ESM.tif]

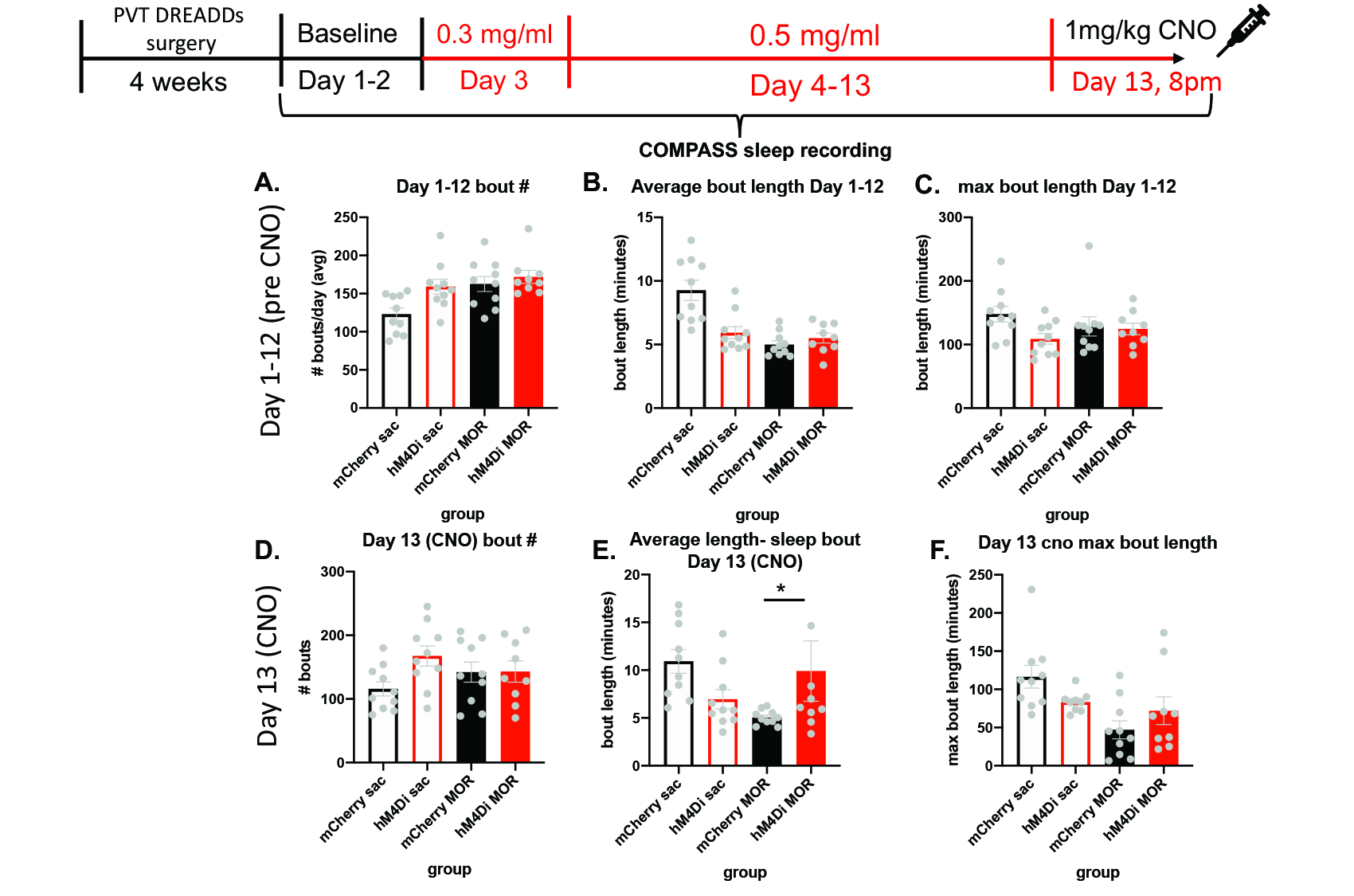

Supplement: Supplementary file 3 — SFigure 2 [file 41398_2023_2382_MOESM3_ESM.tif]

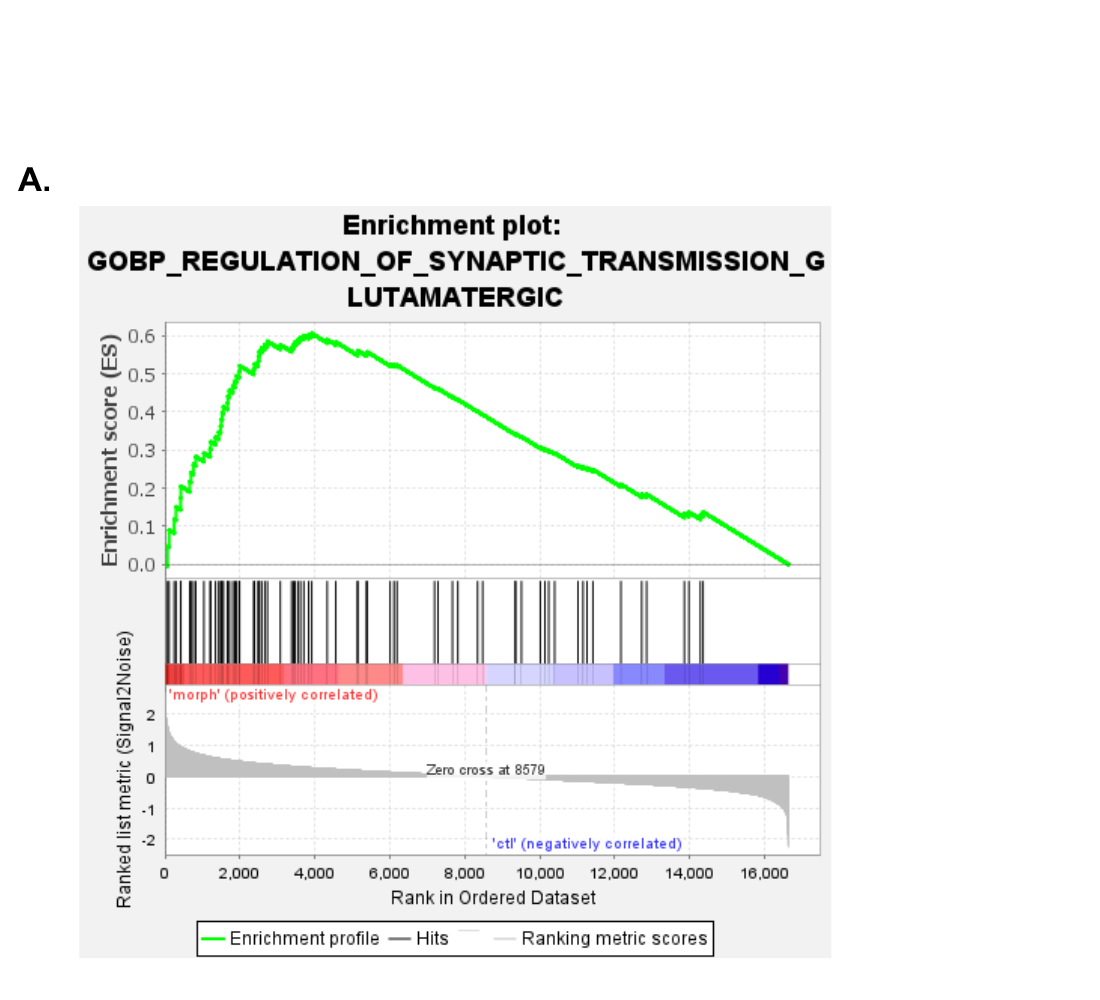

Supplement: Supplementary file 4 — SFigure 3 [file 41398_2023_2382_MOESM4_ESM.tif]
